# Modification of *S. cerevisiae* Growth Dynamics Using Low Frequency Electromagnetic Fields in the 1-2 kHz Range

**DOI:** 10.1155/2015/694713

**Published:** 2015-07-28

**Authors:** Ján Barabáš, Roman Radil, Ivona Malíková

**Affiliations:** University of Žilina, Univerzitná 1, 010 26 Žilina, Slovakia

## Abstract

This paper details our further experiments pertaining to the influence of low frequency electromagnetic fields (LF EMF) on the growth dynamics of two wild-type *Saccharomyces cerevisiae* strands. We opted to explore frequencies beyond the usual 50–60 Hz range, motivated by the ion parametric resonance theory and several studies which discovered and recorded endogenous biosignals in various *Saccharomyces cerevisiae* strands in the 0.4–2.0 kHz frequency range, most probably stemming from microtubules. Both yeast strands used in our experiments have been subjected to continuous 66-hour session of LF EMF exposure (frequencies 1.2, 1.4, 1.6, 1.8, and 2.0 kHz; average magnetic flux density 2.43 mT) under identical ambient conditions. Experiment results indicate a frequency-dependent proliferative response of both yeast strands.

## 1. Introduction

Weak electromagnetic fields, occurring naturally in our environment and present since the beginning of Earth, are progressively being complemented by numerous artificial sources, stemming mainly from the requirements of our modern lifestyle. However, one could argue that these secondary sources of low frequency electromagnetic field (LF EMF) are in sharp contrast with the relatively electromagnetically free environment in which living organisms developed in and adapted to over the course of time. Various scientific studies suggest the presence of an endogenous low frequency electromagnetic field in living organisms around the 0.4–2.0 kHz region [1–5] and thus a valid question arises whether external sources (stimuli) of these frequencies could influence the underlying biological processes. The research presented in this paper therefore focuses on investigation of the proliferative response of biological samples exposed to time-varying LF EMF at frequencies in the 1.2–2.0 kHz range, instead of the traditionally explored 50/60 Hz power line frequencies. Furthermore, our research intends to complement at least few of the hypotheses about the mechanisms of LF EMF exposure at the cellular level. The reason for the mentioned investigation lies in the desire to understand how biological cells can be influenced by means of external LF EMF stimuli. Once this relationship is discovered and clearly described, scientists will have the possibility to control the cellular life using electromagnetic fields in the predictable way and will therefore be able to either inhibit the proliferation process or, on the contrary, support faster cell division in the process of various injuries healing.

## 2. Materials and Methods

**Figure 1:**
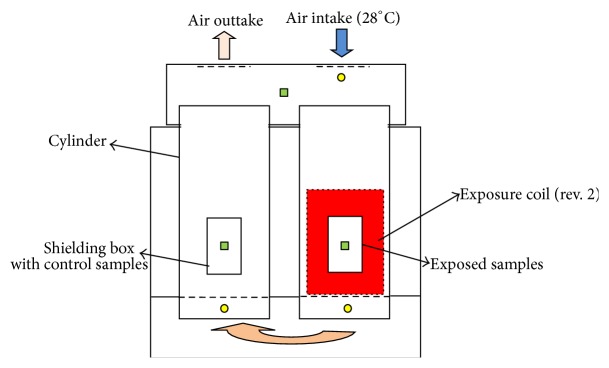
Double-chamber incubator schematic.

The experimental setup consisted of a double-chamber incubator in which Petri dishes with yeast samples were deposited. The purpose of the incubator was to maintain equal temperature of both exposed and control samples during the exposure. As per Figure 1, air was passed through both chambers to prevent possible coil heating and temperature was evaluated at three locations (square markers). The temperature inside the incubator was maintained at a stable 28°C using external air heating device for both the control and the exposed samples throughout the whole duration of the experiment.

**Figure 2:**
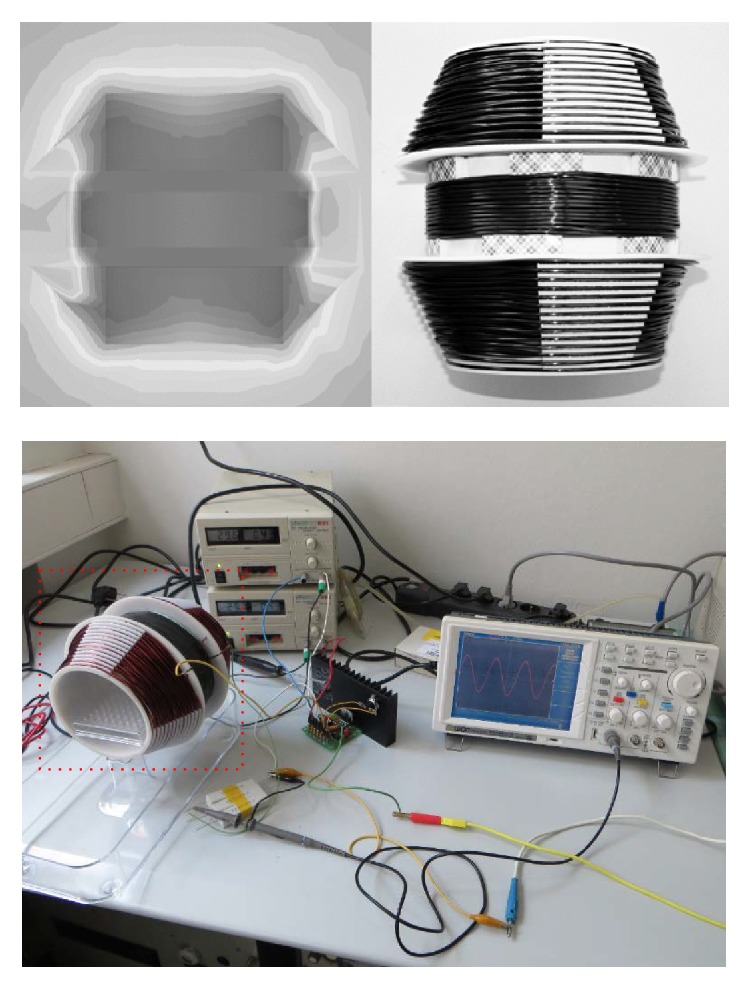
Exposure coil (second revision).

**Figure 3:**
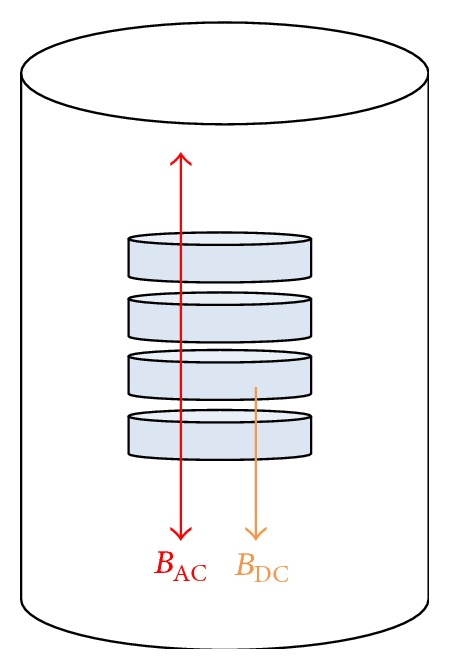
Parallel arrangement of generated *B*_AC_ and static *B*_DC_.

The previously designed exposure coil [6] was replaced by a new construction, shown in Figure 2. The system was designed to assure better homogeneity within the exposed volume (max. 5% magnetic flux density variation within the exposed volume) and thus further verify the reproducibility of previously observed bioeffects. The proposed system was made of appropriate diameter so as to allow the Petri dishes to be placed inside the coil cavity. The copper enameled wire of 1.8 mm in diameter was wound manually using about 100 m of the said material with a resulting inductance *L* = 5.346 mH. The magnetic flux density and homogeneity were first verified numerically using Opera (Cobham plc) and then experimentally using flux gate sensors placed in specific locations along the coil system center axis and also by a commercial electromagnetic field analyzer (Spectran NF 5035, Aaronia AG). Due to the constant flow of air in both chambers no heat elevation was recorded in the coil cavity. All experiments used a parallel configuration of static Earth magnetic field (*B*_DC_, assuming dominant vertical component, as calculated per National Geophysical Data Center magnetic field calculator and measured by Spectran NF 5035) and time-varying *B*_AC_ generated electromagnetic field, as per Figure 3.

**Figure 4:**
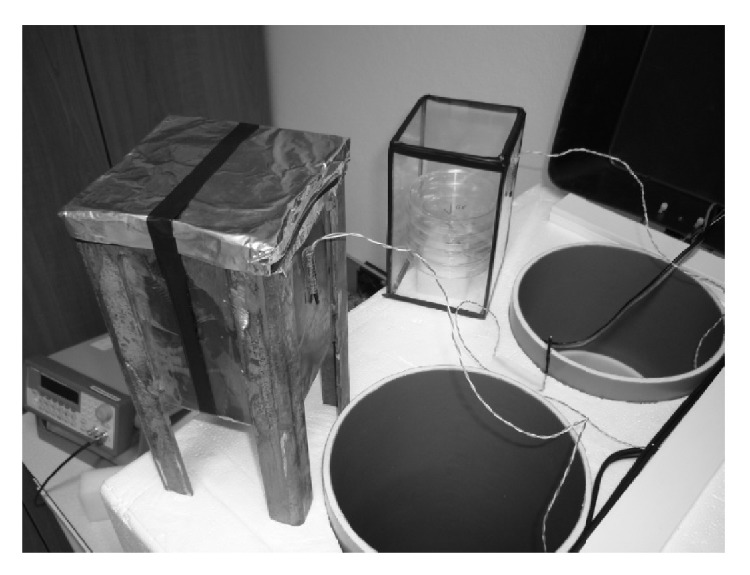
Control samples (left, shielded) and exposed samples (right, unshielded) holders.

Both exposed and control Petri dishes were kept in sample holders (Figure 4) to prevent possible desiccation due to the passing air. The control samples were housed in a mu-metal shielding box and the measured magnetic flux density variation inside the shielding box was on average six orders of magnitude lower than that in the coil center. Both shielding boxes included hydrosensitive paper cards in order to evaluate humidity after experiment.

## 3. Exposure Protocol and Evaluation

We opted to use two different wild type yeast strands (WT1 and WT2) in order to verify our hypothesis of frequency-dependent response. Both sample sources were kept under identical storage conditions and were biologically active at the time of inoculation. Experiments were conducted twice for each frequency (1.2, 1.4, 1.6, 1.8, and 2.0 kHz; 16 Petri dishes per frequency) and yeast strand so as to assure statistically sound results. Each exposure session consisted of eight inoculated Petri dishes: four control and four exposed. The agar used was chloramphenicol yeast glucose agar (GKCH), manufactured by Imuna, Šarišske Michaľany, Slovakia, which is used for cultivation of yeasts and molds. The purity of the used materials and inoculation environment was verified in every experiment by evaluating Petri dish containing pure agar only.

The incubator unit was left running two hours prior to experiment start in order to assure equal and stable temperature of both control and exposed sample housings. The experiment commenced upon loading of all inoculated Petri dishes and was executed for 66 consecutive hours. The harmonic driving signal (1.2, 1.4, 1.6, 1.8, and 2.0 kHz) was generated using a signal generator (Agilent 33220A, Agilent Technologies, Inc.) and amplified using a linear amplifier (Hubert A1110-05, Dr. Hubert GmbH). The measured magnetic flux density within the exposed volume varied between 2.37 and 2.49 mT. No local heating of the exposed samples was recorded in any of the experiments.

**Figure 5:**
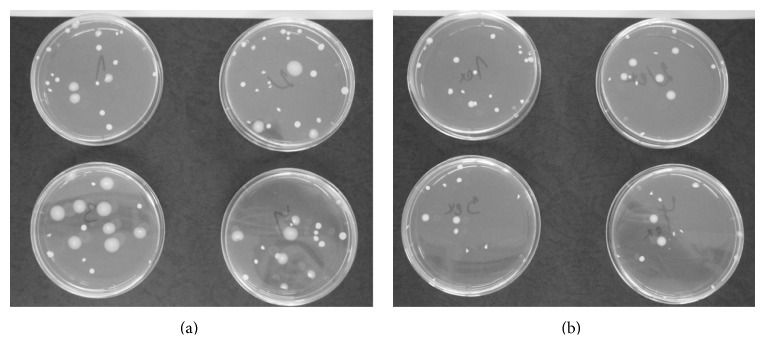
Control (a) and exposed (b) Petri dishes inoculated with WT1 yeast (1.6 kHz, 2.43 mT, and one 66-hour session).

Exposed Petri dishes were positioned in such a way that maximum magnetic flux density was achieved along the center axis. A total of 20 sessions were executed and results were processed using the open-source software OpenCFU (http://opencfu.sourceforge.net/) and our own MATLAB script. For the statistical analysis of the results Student's paired *t*-test was used. Two quantities were observed after exposure: the number of colony forming units (CFU counts) and growth area evaluation (growth dynamics), represented by the area that the said colonies take up on Petri dishes (Figure 5).

## 4. Experiment Results

**Figure 6:**
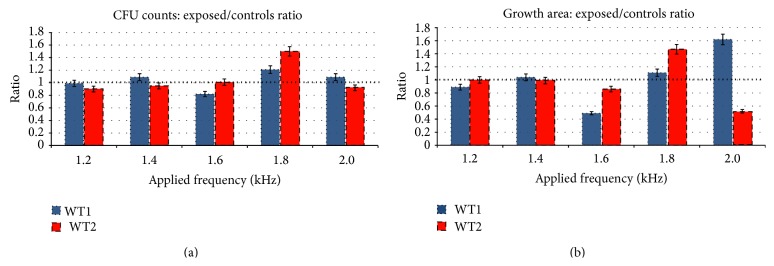
CFU counts (a) and growth area (b) represented as ratios.

Results obtained after exposure have been processed, statistically verified using Student's paired samples *t*-test (Table 1, statistically insignificant results are omitted), and are graphically presented in Figure 6. We opted to represent the data as the ratio between exposed (exp.) and control (cont.) samples for both evaluated quantities: CFU counts and growth area (dynamics). Each exposure was performed twice per frequency and strand type, totaling 160 Petri dishes (excluding the Petri dishes used for purity testing of materials and inoculation procedure). Of interest are especially the growth area ratios for frequencies 1.6 kHz/2.0 kHz for WT1 strand and 1.8 kHz/2.0 kHz for WT2 strand.

## 5. Discussion and Conclusion

In view of currently published mechanisms of action [7–17] we hypothesize that the observed effects can be best quantified using the ion parametric resonance (IPR) theory or via microtubules.

The IPR theory deals with mechanisms of exogenous LF EMF action to ions bound to specific protein locations on cells membranes. These bound ions are responsible for ion channel opening and/or closing and thus for control of ion influx and efflux through the cell membrane, which leads to membrane voltage changes and specific proliferative response. Basically, the theory assumes that parallel combination of static and time-varying LF EMF could affect the bound ions, so that the proliferative behavior of the cell could be controlled in a predictable way.

At this point it is important to note that previously published IPR experiments relied on the application of low frequency electromagnetic fields around the 50 Hz frequency and microtesla range. We adapted a similar approach but considered fields in the millitesla range, thus requiring higher frequencies. These were calculated based on a modified equation for determining the bioactive frequency of Ca^2+^ ions, originally proposed by Lednev and identical to the cyclotron resonance frequency equation, wherein we considered the magnetic flux density *B*_AC_ generated by our exposure coil (as opposed to the static *B*_DC_ considered in the general equation):

(1) $$\begin{array}{c} f_{c\left({Ca}^{2+}\right)}=\frac{q}{2\pi m}B_{AC}. \end{array}$$

The second theory of microtubules stems mainly from experiments conducted by Pokorný et al. Microtubules are cytoskeleton polymers consisting of numerous *α* and *β* tubulin subunits and have an electric dipole moment [18, 19]. They are involved in various cellular processes, of which cell division is the most important in our considerations, and several studies have attributed significantly enhanced levels of electromagnetic activity during the budding phase of yeast cells to microtubules. Assuming that the endogenously generated electromagnetic fields play a role in the cell division process (in addition to chemical pathways), we may consider that the application of exogenous electromagnetic field within the frequency range generated by microtubules might cause signal interference and disruption of the underlying physiological functions. Being frequency-selective, we can achieve the desired effect (proliferative or antiproliferative) by varying the applied frequency. However, other parameters, such as signal shape (harmonic, pulsed, and arbitrary shape) and/or magnetic flux density, might influence the end response, and different response is expected from other organism species.

To conclude, the results of our experiments hint at possible frequency-dependent proliferative response and our findings confirmed previously published results in [6]. The observed biological effects can be explained by either of the mentioned theories. On the other hand, it seems that there are still some uncertainties regarding the precise quantification of observed effects. For example, the magnetic flux density of time-varying LF EMF used for irradiation of experimental biological samples was 10^3^ times higher than the one of static EMF, which could be hypothetically neglected, and the observed effect could be judged as a result of time-varying exposure. To this end, further experiments are necessary to confirm or rule out the above discussed theories.

## Figures and Tables

**Table 1:** Statistical analysis at prominent bioactive frequencies, Student's paired samples *t*-test, and *α* = 0.05.

Strand	Frequency	Evaluated quantity	Mean value	Variance	*p* (two-tail)
WT1	1.6 kHz	CFU counts	5.250	2.375	<0.001
WT1	1.6 kHz	Growth area	5872	1899	<0.001
WT1	2.0 kHz	Growth area	3233	2214	0.010
WT2	1.8 kHz	CFU counts	5.875	2.949	<0.001
WT2	1.8 kHz	Growth area	2219	1768	0.009
WT2	2.0 kHz	Growth area	6030	2335	<0.001
